# Integrative analysis of potential diagnostic markers and therapeutic targets for glomerulus-associated diabetic nephropathy based on cellular senescence

**DOI:** 10.3389/fimmu.2023.1328757

**Published:** 2024-01-08

**Authors:** Donglin Sun, Shuqi Wei, Dandan Wang, Min Zeng, Yihao Mo, Huafeng Li, Caixing Liang, Lu Li, Jun Wei Zhang, Li Wang

**Affiliations:** ^1^ Department of Urology, Shenzhen Hospital, Southern Medical University, Shenzhen, China; ^2^ Center for Cancer and Immunology Research, State Key Laboratory of Respiratory Disease, Guangzhou Medical University, Guangzhou, China; ^3^ Department of Nephrology, Shenzhen Traditional Chinese Medicine Hospital, Shenzhen, Guangdong, China; ^4^ Nephrology Department, Affiliated Hospital of Southern Medical University: Shenzhen Longhua New District People’s Hospital, Shenzhen, China; ^5^ Publicity Department, The Second Affiliated Hospital of Southern University of Science and Technology, Shenzhen, China

**Keywords:** glomeruli, diabetic nephropathy, cellular senescence, molecular docking, diagnostic marker, therapeutic targets

## Abstract

**Introduction:**

Diabetic nephropathy (DN), distinguished by detrimental changes in the renal glomeruli, is regarded as the leading cause of death from end-stage renal disease among diabetics. Cellular senescence plays a paramount role, profoundly affecting the onset and progression of chronic kidney disease (CKD) and acute kidney injuries. This study was designed to delve deeply into the pathological mechanisms between glomerulus-associated DN and cellular senescence.

**Methods:**

Glomerulus-associated DN datasets and cellular senescence-related genes were acquired from the Gene Expression Omnibus (GEO) and CellAge database respectively. By integrating bioinformatics and machine learning methodologies including the LASSO regression analysis and Random Forest, we screened out four signature genes. The receiver operating characteristic (ROC) curve was performed to evaluate the diagnostic performance of the selected genes. Rigorous experimental validations were subsequently conducted in the mouse model to corroborate the identification of three signature genes, namely LOX, FOXD1 and GJA1. Molecular docking with chlorogenic acids (CGA) was further established not only to validate LOX, FOXD1 and GJA1 as diagnostic markers but also reveal their potential therapeutic effects.

**Results and discussion:**

In conclusion, our findings pinpointed three diagnostic markers of glomerulus-associated DN on the basis of cellular senescence. These markers could not only predict an increased risk of DN progression but also present promising therapeutic targets, potentially ushering in innovative treatments for DN in the elderly population.

## Introduction

Diabetic nephropathy (DN), a leading death cause of end-stage kidney disease, is responsible for 30%-40% of cases among diabetic patients (1). The global prevalence of DN is on the rise, dramatically impacting a large proportion of diabetics (2, 3). DN is characterized by structural and functional alternations in the kidney’s glomeruli, the primary units tasked with purifying the blood by filtering out waste and excess fluids. The glomerular damage, manifested as thickening of the glomerular basement membrane and glomerular cell hypertrophy, results in proteinuria, ultimately in turn contributing to the pathogenesis of DN (4, 5).

Cellular senescence, characterized by the permanent cessation of cell proliferation, is intricately linked to the progression of chronic kidney disease (CKD) and acute kidney injuries (6). As highlighted by Coresh et al., 15-38% of individuals aged over 65 suffer from CKD and the percentage rises to 50% for those over 85 years (7). The development of age-related kidney diseases in diabetic is closely related to glomerular lesions, such as glomerulosclerosis and podocyte loss (8, 9). The macrostructural changes on aging kidney including decreased cortical volume and increased surface roughness, which correspond to the typical microstructural features of glomerulosclerosis (10).

Accumulating evidence implicated that DN is highly associated with accelerated senescence in podocytes and mesangial cells of the glomeruli (11–13). However, it is essential to recognize that cellular senescence is a multifaceted process shaped by various factors, and its precise impact on DN warrants deeper investigation. In this study, we employed a synergistic approach combining bioinformatics with machine learning to uncover significant genes connected with cellular senescence and glomerulus-associated DN. Subsequent experimental validations pinpointed three signature genes. Therapeutically targeting cellular senescence and its associated pathways within the glomeruli may offer a promising avenue to mitigate or even halt the progression of DN.

## Materials and methods

### Data acquisition

**Figure 1:**
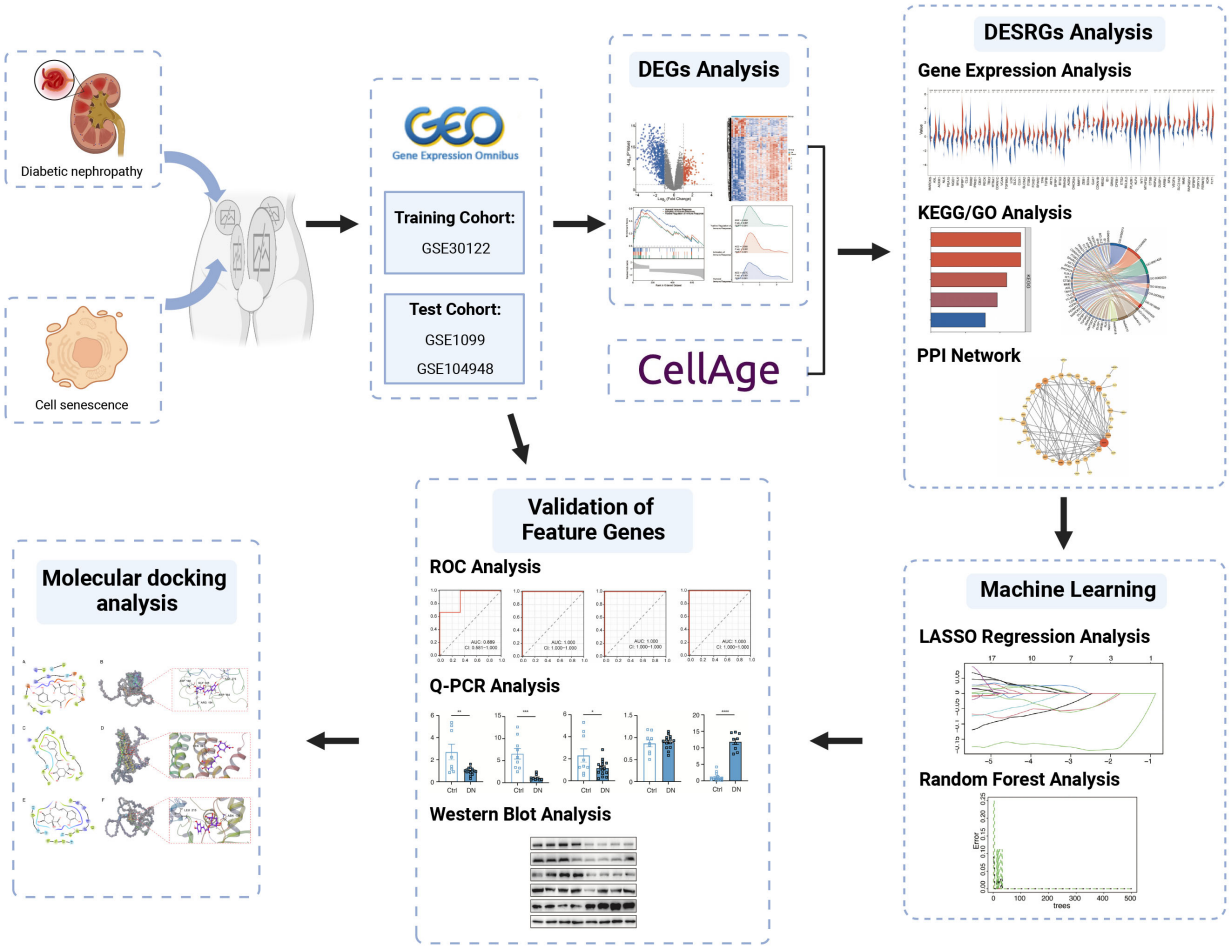
The flowchart of this study.

The microarray datasets of glomeruli-associated DN samples and normal control glomeruli samples were systematically extracted from GEO (https://www.ncbi.nlm.nih.gov/geo/). The selection criteria included: 1) Homo sapiens; 2) expression profiling by array; 3) the experiment included patients with DN and controls; 4) availability of data for glomerulus. GSE30122 dataset consisting of 9 DN samples and 26 control samples was ultimately used as the training cohort, while GSE1009 and GSE104948 datasets were used as the test cohort to verify candidate genes. Meanwhile, a total of 866 cellular senescence-related genes (SRGs) were retrieved and downloaded from CellAge database (**Supplementary Table S1**) (14). The details of these datasets are shown in **Table 1**. The analytical flowchart of this study is illustrated in **Figure 1**.

**Table 1:** Detailed dataset information.

DataSets	Platforms	Sample Size	Organism	Tissue Subregion
GSE30122	GPL8300	9 DN vs. 26 normal	Homo sapiens	glomerulus
GSE1009	GPL20301	3 DN vs. 3 normal	Homo sapiens	glomerulus
GSE104948	GPL22945	7 DN vs. 18 normal	Homo sapiens	glomerulus
Cellular senescence genes	CellAge	886	Homo sapiens	_

### Identification of differentially expressed genes

R statistical software was employed to normalize and analyze the raw microarray data. The analysis of differentially expressed genes (DEGs) between DN patients and controls were conducted by the “limma” package in R software with cut-off values of p< 0.05 and |log2FC|> 1. Probes without matching gene symbols were excluded, and only the probes with the highest signal values for the same molecule were retained. DEGs were visualized in volcano maps and heat maps by conducting R packages “ggplot2” and “ComplexHeatmap” respectively. Gene set enrichment analysis (GSEA) was performed on the DEGs to explore biological signaling pathways by R package “clusterProfiler”. The gene set “c5.go.all.v7.5.1.symbols.gmt” derived from the Molecular Signatures Database was chosen as reference. FDR <0.25 and the p. adjust value <0.05 were considered as statistically significant. Venn diagrams facilitated the screening intersection of DEGs and cellular senescence-associated genes, yielding the final DEGs associated with senescence (hereinafter referred to as DESRGs).

### Function enrichment analysis of cellular senescence-associated DEGs

We deployed “ggplots”, “stats” and “car” packages in R software alongside the Mann Whitney U test to depicit the expressions of DESRGs between DN patients and controls. GO and KEGG enrichment analysis were processed via R package “clusterProfiler” with a threshold p-value of < 0.05 to determine the biological functions of the genes and relevant pathways. Through STRING online database (http://string-db.org), the PPI network was constructed with a combined score >0.4 to evaluate the protein-protein interactions of DESRGs (15). Software Cytoscape facilitated PPI network analysis and visualization.

### Screening of senescence-related signature genes of DN by machine learning

The LASSO regression and Random Forest (RF) methods were adopted to determine hub genes from the 62 DESRGs via “glmnet” and “randomForest” packages respectively (16, 17). Marker genes were selected from variables corresponding to the value of the penalty parameter lambda.min by using 10-fold cross-validation in LASSO regression analysis. The RF model is an ensemble machine-learning algorithm for prediction (18). We set the number of decision trees to 500 and used parametric optimization to find the optimal parameters as “mtry=7”. Genes overlapping between the LASSO model and the top 20 genes from the RF model (with MeanDecreaseGini value >0.2) were defined as signature genes.

### Validation of signature genes

The receiver operating characteristic (ROC) curve was employed to evaluate the predictive accuracy of signature genes by the “pROC” R package in both training cohort (GSE30122) and test cohorts (GSE1009 and GSE104948). Visualization of results was implemented with the ggplot2 package and the genes with AUC > 0.7 were considered diagnostic.

### Construction of the mouse model

Mice were randomly allocated into two groups: (i) a control group with normal blood glucose levels (Control group, n = 9-15), and (ii) streptozotocin (STZ)-induced diabetic mice (DN group, n = 9-15). Following a 12-hour fast, STZ (Sigma Chemicals, St. Louis, MO) was administered intraperitoneally (IP) at a dose of 50mg/kg, dissolved in 100mM, for five consecutive days. Diabetes was induced in mice aged 6-8 weeks, weighing 18-20g. The control group received citrate buffer (pH 4.5) for five consecutive days. Control mice received an equivalent volume of citrate buffer. After two weeks, blood was collected via submandibular vein puncture, and fasting blood glucose levels were measured using the HemoCue B-Glucose kit (HemoCue AB, Angelholm, Sweden). Mice with fasting blood glucose levels ≥ 12mmol/L were considered diabetic and included in further studies. After 20 weeks of STZ treatment, final body weight and kidney weight were measured. Blood samples were collected before euthanizing the mice under anesthesia. Some mice in various groups did not survive the induction process. All procedures were conducted following the Guide for the Care and Use of Laboratory Animals of the National Institutes of Health, as well as the Animal Welfare Act guidelines.

### Measurements of blood and urinary parameters

Serum and urine creatinine values were quantified employing high-performance liquid chromatography (HPLC, Beckman Instruments, Fullerton, CA, USA). The urinary albumin value was determined using an immunoassay method (Bayer, Elkhart, IN, USA). The urinary albumin-to-creatinine ratio (UACR) was computed as the quotient of urine albumin and urine creatinine concentrations (μg/mg). All assays were executed according to the manufacturer’s protocols.

### Analysis of quantitative real-time PCR

RNA was extracted from diabetic kidney tissue using the TRIzol reagent. The extracted RNA was converted into cDNA while removing possible genomic DNA contamination using the PrimeScript™ RT reagent Kit with gDNA Eraser. Real-time RT-PCR was performed using the Q7 RT-PCR Detection System and SYBR^®^ premix Ex Taq. Relative quantification (RQ) was calculated based on the 2^(-ΔΔCt) method, normalizing outcomes to β-actin mRNA as the control. The primer sequences used in the experiments are listed in **Supplementary Table S2**. The overarching objective of this experiment was to gauge specific gene expression levels in diabetic kidney relative to various samples.

### Western blot

Diabetic kidney specimens were rinsed with pre-cooled PBS before homogenization. The homogenized tissues were then lysed in RIPA buffer (Beyotime, P0013F) containing protease inhibitor mixture (Roche Diagnostics, 4693116001), phosphatase inhibitor mixture (Roche Diagnostics, 4906845001), and PMSF (Beyotime, ST506). After a 30-minute ice bath incubation, the lysates were centrifuged at 13,000g for 15 minutes and the supernatant were stored at -80°C. Total protein concentration was estimated using the BCA Protein Assay Kit (Thermo Fisher Scientific, 23225). Protein samples (10-20μg) were diluted with 5x loading buffer, heated in a metal bath for 10-15 minutes, separated by 12.5% SDS polyacrylamide gel electrophoresis (SDS-PAGE), and then transferred to PVDF membranes (Millipore) for antibody probing. Immunoreactive proteins were detected using an enhanced chemiluminescence detection system (Millipore). We deployed a gamut of primary antibodies including Anti-LOX (CST, #58135), Anti-FOXD1 (Invitrogen, #PA5-35145), Anti-GJA1 (CST; #83649), Anti-BTG3 (Sangon Biotech; D220325) and Anti-GAPDH (KANGCHEN, KC-5G4). The secondary antibodies used were peroxidase-conjugated goat anti-mouse IgG (H+L) (33201ES60, Yeasen) and peroxidase-conjugated rabbit anti-goat IgG (H+L) (33701ES60, Yeasen). This Western blot procedure was conducted to discern specific protein expression levels in diabetic kidney samples, leveraging targeted antibodies for detection.

### Molecular docking analysis

Eucommia ulmoides Oliver (EUO), one of the traditional Chinese medicine, is renowned for its effective treatment of kidney deficiency, lipid reduction and anti-obesity, attributable to the high concentrations of chlorogenic acids (CGA), quercetin, iridoid and α-linolenic acid in its leaves and bark (19, 20). CGA, also abundant in coffee beans, was reported to have substantial biological activities possibly mediating coffee’s beneficial impacts on glucose regulation and the development of type 2 diabetes (21). Zheng et al. presented that CGA have a positive effect on senescence delays and lifespan extension of Caenorhabditis elegans (22). However, a comprehensive review of the effects and mechanisms of CGA is currently unavailable. Therefore, our investigation sought to demystify the therapeutic potentials of CGA through the molecular docking.

The molecular structure of CGA was derived from the PubChem database (https://pubchem.ncbi.nlm.nih.gov/), processed by the LigPrep module in Schrödinger software to generate the 3D conformations (23). AlphaFold2 (https://alphafold.ebi.ac.uk/) was utilized to predict the crystal structures of potential proteins (24). The obtained protein crystals were processed using the Protein Preparation Wizard of Schrödinger, including protein preprocess, regenerate states of native ligand, H-bond assignment optimization, protein energy minimization and remove waters. SiteMap and Receptor Grid Generation modules were applied to predict active sites of targeted proteins (25). We then performed the extra-precision docking (XP docking) of ligand compound CGA and active sites of the potential proteins. The lower XP Gscore represents the lower binding free energy between the compound and the protein, which implies higher binding stability (26). Molecular mechanics generalized Born surface area (MM-GBSA Analysis) was used to confirm the ligand-protein interaction and the binding free energy (27).

### Statistical analysis

R software (version 4.2.1) and GraphPad Prism version 8.1 were applied in this study for all statistical analysis and graph visualization. A p-value of less than 0.05 was considered statistically significant.

## Results

### Screening of DEGs in DN glomeruli cells and enrichment analysis

**Figure 2:**
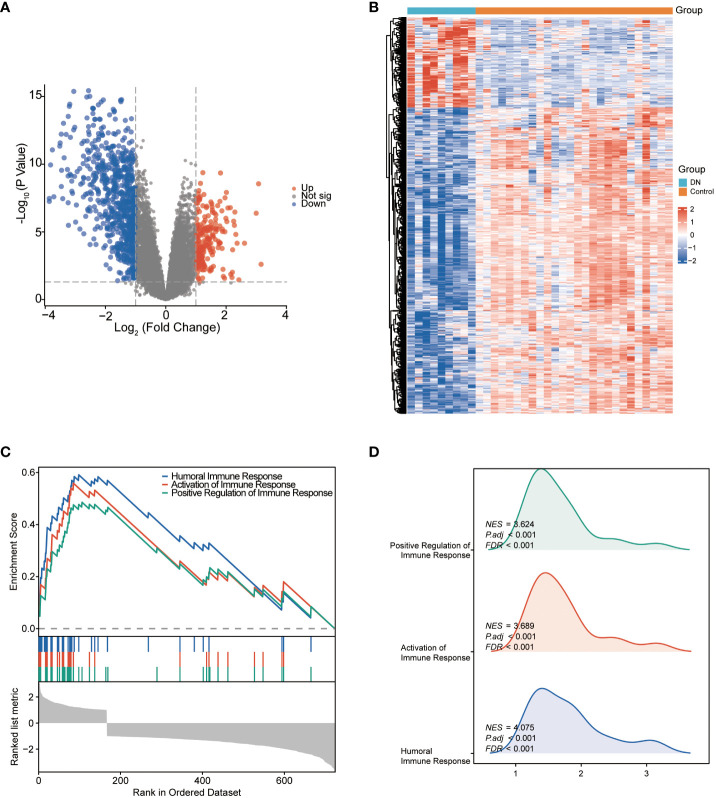
The identification and the enrichment analysis of DEGs. **(A)** Volcano plot of DEGs between the DN and control groups. Red dots represent upregulated genes and blue dots represent downregulated genes. **(B)** Heatmap of DEGs. The horizontal axis shows a sample type, and the vertical axis displays the difference in expression between genomes. **(C)** Gene set enrichment analysis for DEGs. **(D)** GSEA ridgeplot that showed NES value of top GO terms.

A total of 756 DEGs were obtained from the comparisons between DN patients and control samples in the training cohort GSE30122, including 172 significantly upregulated and 584 significantly downregulated genes. Volcano plot and heatmap were utilized to depict these DEGs (**Figures 2A, B**). GSEA results (**Figure 2C**) revealed that the primary involvement of DEGs was in the immune responses processes. The top three correlations enriched depending on normalized enrichment score (NES) values were humoral immune response, activation of immune response, and positive regulation of immune response (**Figure 2D**).

### Identification and integrative analysis of DEGSRs

**Figure 3:**
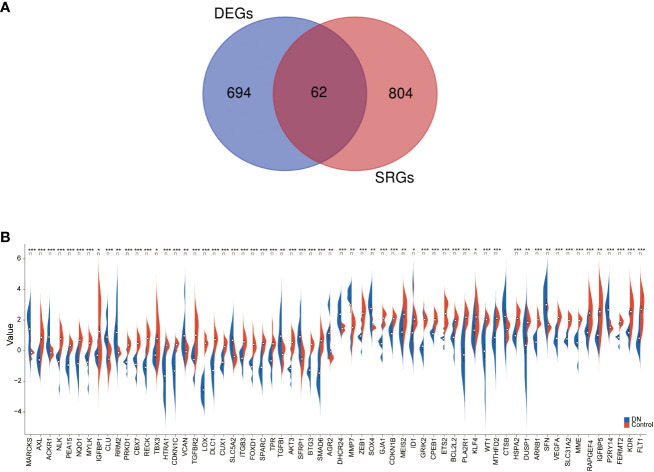
The identification and mRNA expression analysis of DESRGs. **(A)** Overlapping genes in DEGs and aging-related genes. **(B)** The difference in the mRNA expression profiling of DESRGs between the DN and control groups. *P < 0.05, **P < 0.01, and ***P < 0.001.

**Figure 4:**
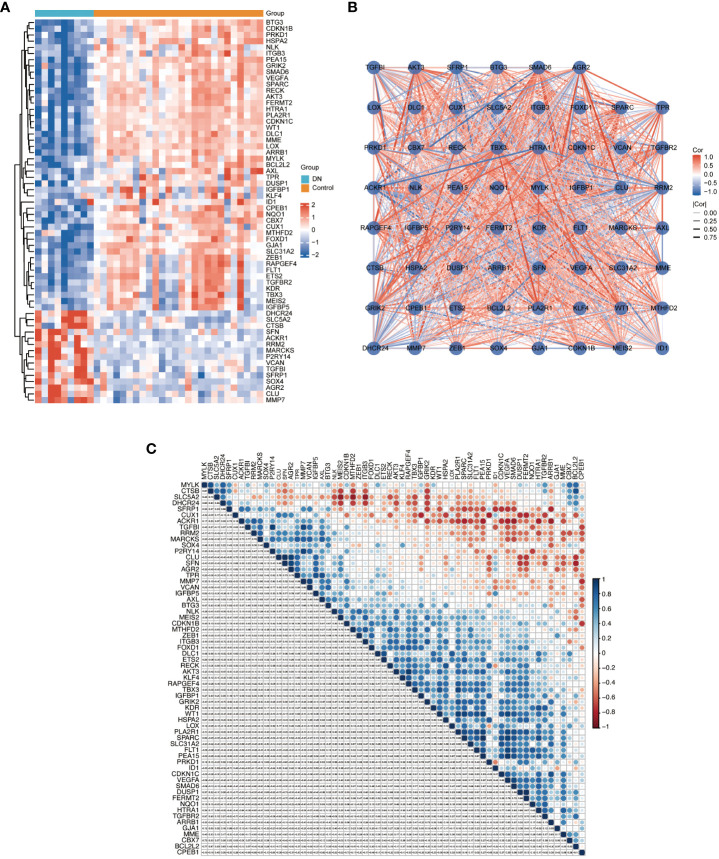
The heatmap and correlation analysis of DESRGs. **(A)** Heatmap to visualize the expressions of DESRGs. **(B)** The correlation network plot of DESRGs in the DN group. **(C)** The correlation heatmap of DESRGs in the DN group. *P < 0.05, **P < 0.01, ***P < 0.001, and ****P < 0.0001.

We applied Venn diagram to determine 62 overlapped genes after intersecting the 756 DEGs with 866 cellular senescence-associated genes derived from the CellAge database (**Figure 3A**). Among these 62 DESRGs, 15 genes were upregulated and 47 genes were downregulated (**Figures 3B**, **4A**). The correlations between DESRGs were demonstrated through Spearman correlation test in **Figure 4B**, suggesting all DESRGs interacted with each other. The color intensity and shape magnitude in **Figure 4C** highlighted the strength and direction of the relationships between each DESRG, with darker colors indicating stronger correlations.

**Figure 5:**
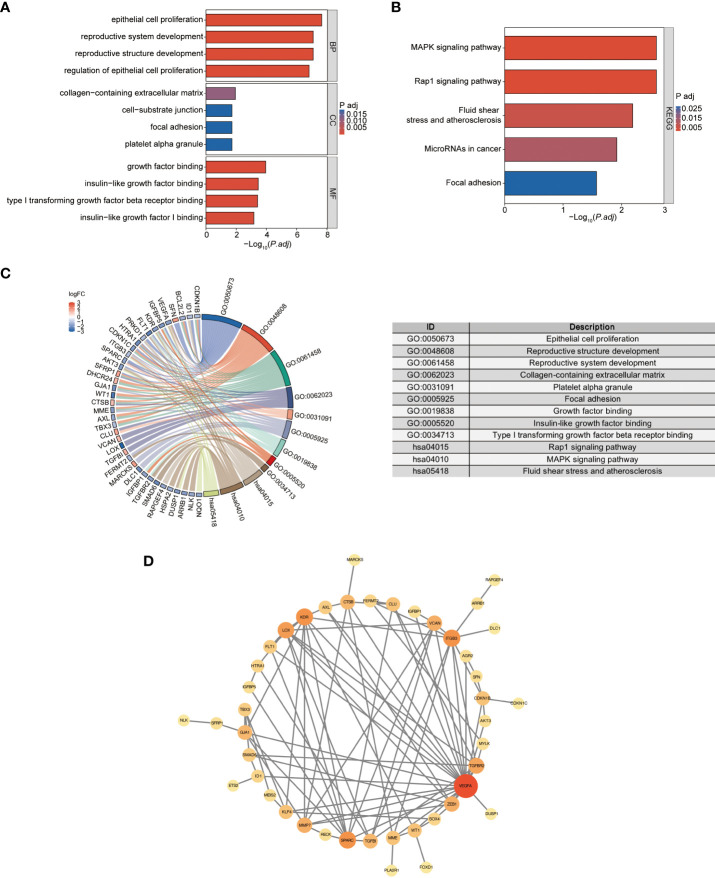
the GO and KEGG analysis of DESRGs and the PPI network construction. **(A)** Biological processes (BPs), cellular components (CCs), and molecular functions (MFs) associated with DESRGs. **(B)** KEGG pathways enriched by DESRGs. **(C)** Chord plot of enriched genes, GO terms and KEGG pathways. **(D)** PPI network of cellular senescence-related DEGs.

To explore the biological functions and enrichment pathways, GO and KEGG analysis were performed. As shown in the results of GO and KEGG analysis (**Figures 5A, B**), the functional enrichments of 62 DESRGs were markedly involved in epithelial cell proliferation, reproductive structure and system development, regulation of epithelial cell proliferation, MAPK signaling pathway and Rap1 signaling pathway. The significantly enriched genes were revealed in **Figure 5C**. The PPI network was constructed through STRING database to further analyze the interactions between 62 DESRGs. A total of 48 nodes and 97 edges were identified (**Figure 5D**).

### Feature genes selections by machine learning

**Figure 6:**
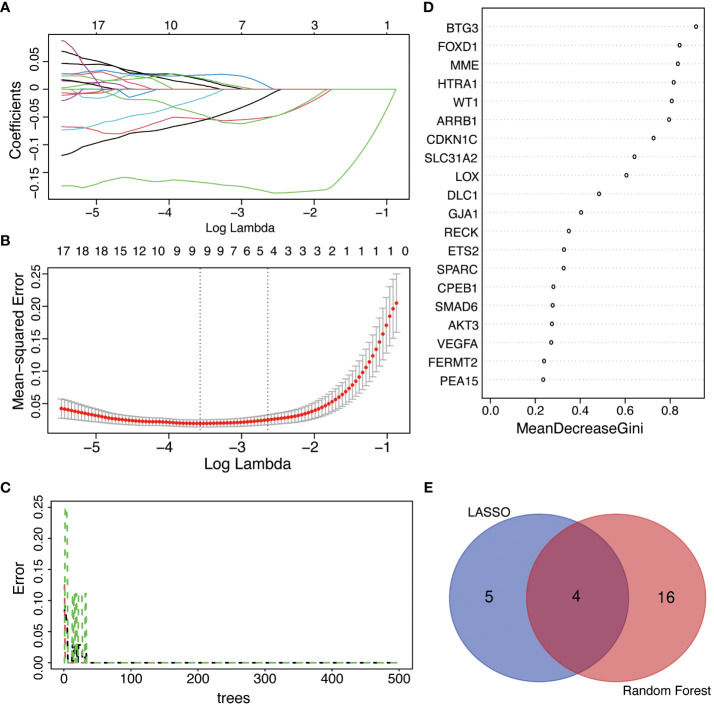
The selection of feature genes by applying machine learning algorithms. **(A)** The coefficient from LASSO regression analysis. Different colors represent different genes. **(B)** Feature genes screened from LASSO algorithm. **(C)** Accuracy of RF algorithm. **(D)** Importance ranking of top 20 genes screened by RF algorithm. Average accuracy decreased in importance and Gini index. **(E)** Venn diagram of the intersection of screening results among LASSO and RF.

LASSO regression analysis was applied among all selected DESRGs to screen out 9 candidate genes, including DHCR24, GJA1, SFN, P2RY14, MARCKS, LOX, FOXD1, TPR and BTG3 (**Figures 6A, B**). Utilizing RF combined with feature selection, 20 genes were ranked based on their relative relevance with an estimated out-of-bag (OOB) error rate of 0% (**Figures 6C, D**). The intersection of the 9 candidate genes from the LASSO analysis with the top 20 feature genes from RF yielded four signature genes, namely BTG3, LOX, FOXD1 and GJA1 (**Figure 6E**).

### Diagnostic significance of signature genes

**Figure 7:**
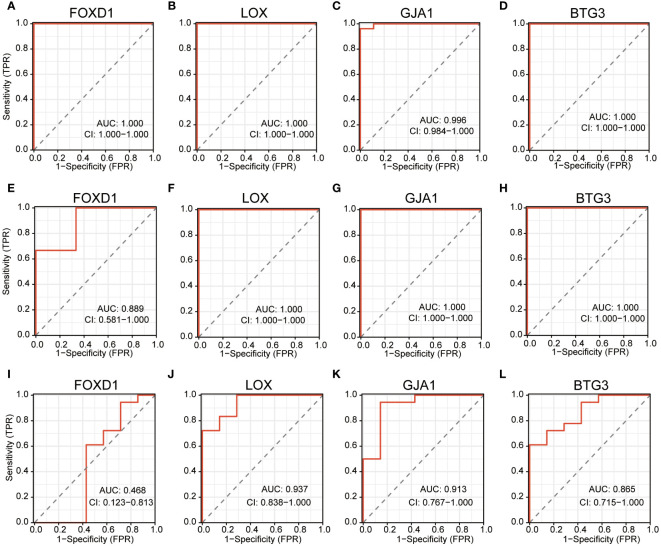
ROC curves estimating the diagnostic performance of the feature genes. **(A–D)** ROC curves of FOXD1, LOX, GJA1 and BTG3 in training cohort GSE30122. **(E–H)** ROC curves of FOXD1, LOX, GJA1 and BTG3 in test cohort GSE1009. **(I–L)** ROC curves of FOXD1, LOX, GJA1 and BTG3 in test cohort GSE104948.

ROC curves were operated in the training cohort to assess the diagnostic potential of signature genes. FOXD1, LOX, GJA1 and BTG3 showcased outstanding diagnostic values for differentiating DN patients from healthy individuals, with AUC values of 1.000, 1.000, 0.996 and 1.000 respectively (**Figures 7A–D**). We also created ROC curves in the test cohorts GSE1009 and GSE104948. In the GSE1009, these four characteristic genes all had significant diagnostic performance with AUC values of 0.889 in FOXD1, 1.000 in LOX, 1.000 in GJA1 and 1.000 in BTG3 (**Figures 7E–H**). In the GSE104948, LOX, GJA1 and BTG3 showed great predictive ability with respective AUC values of 0.937, 0.913, and 0.865, while FOXD1 had AUC of 0.468 (**Figures 7I–L**). These broadly similar findings in both training cohort and test cohorts suggested these four feature genes as potential diagnostic markers for DN.

### Validating the identified feature genes in a murine DN model

**Figure 8:**
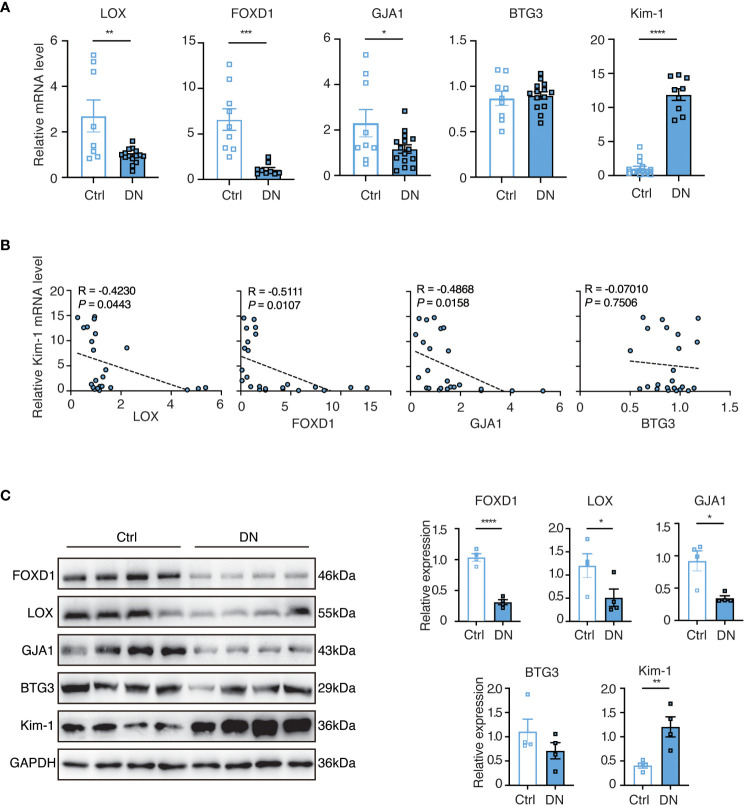
The validation of selected feature genes in DN mouse experiments. **(A)** Quantitative real-time PCR (q-PCR) analysis of mRNA expression levels of the identified genes in homogenized kidney tissues from the DN mouse model (n = 9-15). Statistical analysis was performed using the t-test to determine significant differences between the control and DN groups. **(B)** Correlative scatterplots illustrating the relationship between the identified key genes in the kidney and the mRNA expression levels of Kim-1, determined by Spearman rank correlation (R). **(C)** Western blot analysis of protein expression levels of the relevant genes in homogenized kidney tissues from the DN mouse model (n = 4). Statistical analyses were conducted using the Student’s t-test to identify significant differences between the control and DN groups.

To further validate our findings, we conducted experiments using a mouse model of DN. In comparison to the controls, the levels of LOX, FOXD1, and GJA1 were decreased while Kim-1 was significantly elevated in the DN mice. However, the expression of BTG3 in the DN mice did not show a significant change (**Figure 8A**). We then performed correlation analysis and observed that LOX, FOXD1, and GJA1 exhibited a negative correlation with Kim-1, while BTG3 showed no significant correlation with Kim-1 (**Figure 8B**). Subsequently, Western blot on diabetic kidney tissue confirmed these findings, indicating that the protein levels of LOX, FOXD1 and GJA1 also significantly decreased while Kim-1 in DN mice increased (**Figure 8C**).

We then assessed renal function in serum and urine samples from the mouse model by measuring serum creatinine (Scr) and the urine albumin-creatinine ratio (uACR) and performed the correlation analysis with the relative expressions of selected feature gens (Relative to GAPDH). **Supplementary Figure S1A** indicated that Scr and uACR in the DN group were significantly higher than in the control group, confirming the successful modeling. LOX and FOXD1 exhibited significantly negative correlations with Scr, while BTG3 and GJA1 demonstrated negative correlations without significance (**Supplementary Figure S1B**). LOX, FOXD1 and GJA1 showed significantly negative correlations with uACR while BTG3 had no significance (**Supplementary Figure S1C**). **Supplementary Figure S1D** depicted the correlation between the quantified protein expression levels of feature genes and Kim-1 (**Figure 8C**) detected in mouse kidney tissue. FOXD1 and GJA1 displayed negative correlations with the relative expressions of Kim-1. These findings were consistent with our initial analysis predictions, suggesting the potential of LOX, FOXD1 and GJA1 as indicators for DN in aging mice. Moreover, these genes might prospectively become therapeutic targets to manage elderly DN patients.

### Validation of the targets using molecular docking

LOX, FOXD1 and GJA1, identified in the mice experiments, were subjected to molecular docking analysis alongside CGA to evaluate their potential therapeutic impacts on aging DN patients. The binding affinity and binding-free energy were gauged using XP Gscore (kcal/mol) and MM-GBSA dG Bind (kcal/mol) metrics respectively, to determine the ligands’ positions. The results of XP Gscore and MM-GBSA dG Bind between CGA and the three key targets were presented in **Table 2**. LOX, FOXD1 and GJA1 were all demonstrated low binding free energy with MM-GBSA dG Bind values of lower than -30 kcal/mol, indicating the strong stability of ligand-protein binding. Among all three key proteins, GJA1 showcased the most stable and sufficient binding with CGA by its lowest XP Gscore of -7.045. FOXD1 followed with an XP Gscore of -6.273, demonstrating its fairly substantial binding stability.

**Table 2:** Results of molecular docking between CGA and key targets.

Compound	Key Target	XP Gscore (kcal/mol)	MM-GBSA dG Bind (kcal/mol)
Chlorogenic acid	GJA1	-7.045	-31.43
	FOXD1	-6.273	-39.63
	LOX	-4.277	-41.82

**Figure 9:**
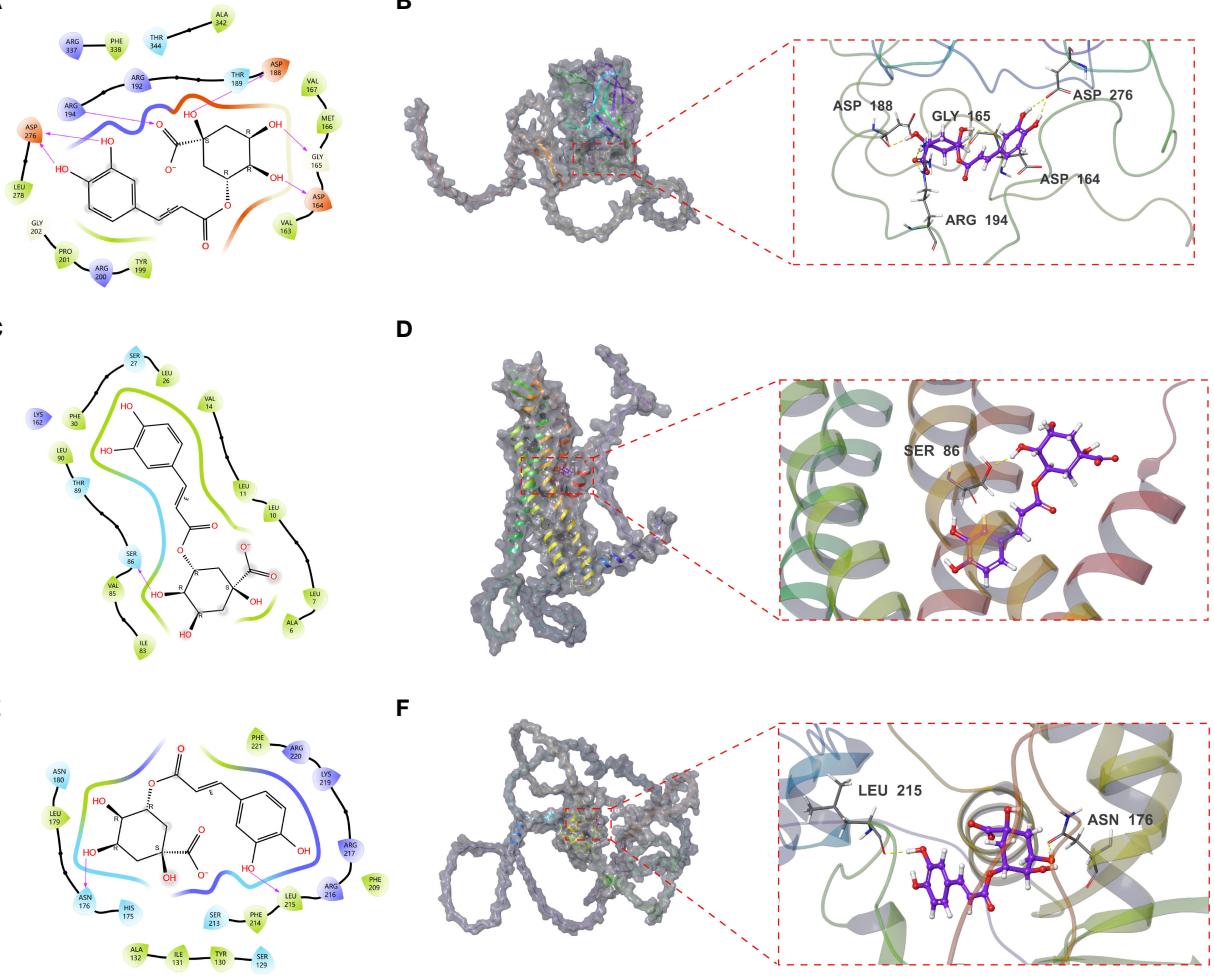
Molecular docking results of CGA interaction with GJA1, LOX and FOXD1. **(A, B)** Molecular docking conformation of CGA interaction with GJA1. **(C, D)** Molecular docking conformation of CGA interaction with LOX. **(E, F)** Molecular docking conformation of CGA interaction with FOXD1.

**Figures 9A, B** depicted how CGA interacted with GJA1 by forming a hydrogen bond with SER86 and establishing hydrophobic interactions with surrounding residues including LEU90, PHE30, LEU26, VAL14, LEU11, etc. The interaction between CGA and FOXD1, where CGA created hydrogen bonds with ASN176 and LEU215, was illustrated in **Figures 9C, D**. CGA was additionally observed to interact with LOX as it formed hydrogen bonds with ASP188, GLY165, and ASP164, along with double hydrogen bonds with both ASP276 and ARG19, as shown in **Figures 9E, F**.

## Discussion

The glomerulus, as the fundamental functional unit of the kidney, filters blood to produce urine. Persistent hyperglycemia and associated metabolic disturbances in DN lead to the excessive formation of the extracellular matrix and glomerular mesangial dilatation, further occluding glomerular capillaries and compromising glomerular functions (28, 29). Recent research emphasized Cellular senescence as a pivotal factor in these pathological developments (30). The occurrence of cellular senescence in glomeruli stems from oxidative stress and inflammation responses, both triggered by the chronic hyperglycemia observed in DN patients (31, 32). There is an increasing interest to comprehensively explore senescent cells as a potential therapy strategy. Consequently, the main purpose of this study is to elucidate the molecular mechanisms of cellular senescence in glomerulus-associated DN, paving the way for identifying innovative therapeutic targets and fostering the development of more efficacious treatments.

From our research, 62 DESRGs were screened out from the combination of aging genes and DN genes in glomeruli with differential expression. Out of these, 15 genes were upregulated and 47 genes were downregulated. GO analysis revealed that DESRGs were prominently enriched in the biological process of epithelial cell proliferation, reproductive system and structure development, all of which can accelerate the onset of CKD (33). KEGG pathway analysis demonstrated that DESRGs were mainly involved in MAPK signaling pathway, which is the core in the regulation of cellular growth and survival, and Rap1 signaling pathway, which modulates cell proliferation and cell differentiation (34, 35). Leveraging two machine learning algorithms, LASSO analysis and RF, we pinpointed four feature genes (FOXD1, LOX, GJA1 and BTG3). Moreover, a mouse model was established to further validate that FOXD1, LOX and GJA1 might serve as potential biomarkers of clinical diagnostic and risk evaluation for aging DN patients.

FOXD1 is required for kidney development, fostering the differentiation of nephron progenitor cells by repressing decorin in the embryonic kidney (36, 37). Humphreys et al. confirmed that FOXD1 may be dynamically regulated during kidney injury and repair in the developing mouse model (38). Previous studies established connections between FOXD1 and kidney tumor development, as well as mitochondrial metabolism (39). FOXD1 was additionally underscored to promote cell proliferation by targeting the sonic hedgehog pathway and cyclin-dependent kinase inhibitors (40). This is particularly relevant since Sonic Hedgehog signaling controls fibroblast proliferation and renal fibrosis. Targeting this pathway may offer therapeutic interventions for DN. Conversely, the downregulation of FOXD1, attributed to YAP deficiency, has been reported to contribute to the early onset of cellular aging. Lentiviral gene transfer of YAP or FOXD1 has demonstrated a mitigating effect on cellular senescence (41). Considering our observation of a significant downregulation of FOXD1 in elderly DN groups, it suggested that FOXD1 may not only play a role in the progression of DN but also potentially accelerate cellular senescence. This intriguing association underscores the multifaceted involvement of FOXD1 in renal health and aging processes.

LOX, an enzyme instrumental in extracellular matrix (ECM) formation and maintenance, achieves this by cross-linking collagen and elastin fibers (42, 43). The excessive accumulation of ECM, which might lead to mesangial matrix expansion and thickening of glomerular basement membrane, signified DN-related pathological shifts (44, 45). Prior research observed a close association between LOX expression and histologic alterations in early-stage DN in type 2 diabetes male ZDF rat models (46). The established implication of LOX in conditions characterized by inflammatory components is well-documented (47). With the mediation of TNF-α, LOX was reportedly downregulated in inflammatory conditions such as diabetes and osteoporosis, thereby suppressing cell growth and reducing the pluripotent cell pool (48). In the present study, we discovered the downregulation of LOX in DN group. It is speculated that the absence of LOX activity in the glomerulus could potentially disrupt the typical remodeling and repair processes within the renal ECM, leading to the promotion of cell senescence. This alteration could ultimately contribute to the structural and functional abnormalities observed in DN.

Another pivotal gene identified was GJA1, encoding for the protein Connexin 43 (Cx43) which forms essential gap junctions for cell cycle regulation, wound healing, and muscle differentiation (49, 50). Cx43 was confirmed to closely associate with the pathogenesis of overt DN. Satriano et al. observed a reduced Cx43 expression in rats with experimentally induced type 1 diabetes using streptozotocin (STZ) treatment (51). Consistent findings were reported by Hu et al. and Zhang et al., who documented decreased Cx43 expression in db/db mice with type 2 diabetes and leptin receptor deficiency in response to elevated glucose levels (52, 53). Moreover, the heightened Cx43 expression demonstrated an enhanced activation of the Nrf2/ARE pathway, offering protection against oxidative stress and subsequently mitigating renal fibrosis in diabetic mice (54). Recent reports in patients with type 2 diabetes mellitus manifested the expression of tight junction markers, including GJA1, was significantly suppressed by hyper-activating Apelin (APLN) peptide and downregulated in diabetic testis (55). In a diabetic mouse model, the downregulation of Cx43 was adequate to disrupt vascular homeostasis and induce apoptosis, contributing to the development of diabetic retinopathy (56). In alignment with these findings, our study unveiled that the downregulation of GJA1 in glomerulus-associated DN, intimately correlated with the occurrence of cell senescence.

CGA, a phenolic compound, boasts antioxidant, anti-inflammatory, anticarcinogenic and antibacterial properties (57–60). Pioneering works by Yun et al. and Jiang et al. disclosed the capacity of CGA to activate Nrf2 and stimulate antioxidant enzymatic activities, thereby counteracting oxidative damage (57, 58). Bao et al. further pointed out that CGA treatment applied its potent antioxidant and anti-inflammatory attributes to protect against DN, predominantly through the modulation of the Nrf2/HO-1 and NF-ĸB pathways *in vitro* and *in vivo* (61). The efficacy of CGA in addressing obesity, reducing fasting plasma glucose and enhancing insulin sensitivity was also well-documented (62, 63). Through the molecular docking analysis in our study, three target proteins (FOXD1, LOX and GJA1) could potentially predict the therapeutic effects of CGA on aging-related DN.

This study has several limitations to be addressed. Firstly, our research findings are based on public databases, lacking detailed clinical information such as severity levels of kidney and survival data. Further investigations with prospective clinical data are essential to corroborate our results. Secondly, our sample sizes were relatively modest, particularly in the training cohort, although external validations were performed to mitigate this limitation. It is possible that additional feature genes contributing to the progression were neglected in our research. Last but not least, additional studies involving animal and human subjects are required to validate these results and investigate the specific roles and mechanisms of aging cells in DN progression.

## Conclusion

In summary, this study illuminated the involvement of cellular senescence in the pathological mechanisms of glomerulus-associated DN at the molecular level. The identification and validation of three signature genes, namely FOXD1, LOX, GJA1, could provide comprehensive insights of the escalating risks of DN progression among the elderly. Their potential therapeutic implications hold the promise of advancing more targeted and effective treatment strategies for DN, a pressing medical challenge in the aging population.

## Data availability statement

The original contributions presented in the study are included in the article/**Supplementary Material**. Further inquiries can be directed to the corresponding authors.

## Ethics statement

Experimental animals were cared for and used in accordance with the Guide for the Care and Use of Laboratory Animals of Rise Mice Biotechnology Co., Ltd. (Zhaoqing, China). The animal experiment of the study was approved by Shenzhen Hospital of Southern Medical University’s Institutional Biomedical Research Ethics Committee. The study was conducted in accordance with the local legislation and institutional requirements.

## Author contributions

DS: Conceptualization, Data curation, Formal analysis, Validation, Writing – original draft, Writing – review & editing. SW: Methodology, Project administration, Validation, Visualization, Writing – original draft. DW: Methodology, Project administration, Writing – original draft, Data curation, Formal analysis. MZ: Formal Analysis, Methodology, Software, Writing – review & editing. YM: Methodology, Writing – review & editing, Data curation, Resources. HL: Methodology, Writing – review & editing, Software. CL: Software, Writing – review & editing, Resources, Supervision. LL: Resources, Visualization, Writing – original draft. JZ: Resources, Supervision, Validation, Writing – review & editing. LW: Writing – review & editing, Conceptualization, Funding acquisition.
