# Supplementary material for: Integrative analysis of potential diagnostic markers and therapeutic targets for glomerulus-associated diabetic nephropathy based on cellular senescence
Source: Front Immunol. 2024 Jan 8;14:1328757. doi: 10.3389/fimmu.2023.1328757 (PMC10881763; doi:10.3389/fimmu.2023.1328757)
Supplement: Supplementary file 1 [file DataSheet_1.pdf]

**Supplementary Materials**

Table S1. cellular senescence-related genes from CellAge database

| Gene symbol | Senescence Effect |
|---|---|
| AAK1 | Induces |
| ABCB1 | Induces |
| ABCC6 | Inhibits |
| ABI3 | Induces |
| ABI3BP | Induces |
| ACER2 | Inhibits |
| ACKR1 | Induces |
| ACLY | Inhibits |
| ADCK5 | Induces |
| AGO2 | Inhibits |
| AGR2 | Inhibits |
| AGT | Induces |
| AHR | Induces |
| AKAP4 | Inhibits |
| AKR1B1 | Inhibits |
| AKT1 | Induces |
| AKT1S1 | Inhibits |
| AKT3 | Inhibits |
| AKTIP | Inhibits |
| ALDH2 | Inhibits |
| ALDOA | Inhibits |
| ALKBH3 | Inhibits |
| ALOX15B | Induces |
| ANAPC1 | Inhibits |
| ANLN | Inhibits |
| ANXA5 | Inhibits |
| APEX1 | Inhibits |
| AR | Induces |
| ARF1 | Induces |
| ARG2 | Induces |
| ARID1A | Induces |
| ARID1B | Induces |
| ARID3A | Inhibits |
| ARID4B | Induces |
| ARPC1B | Induces |

| | |
|---|---|
| ARRB1 | Induces |
| ASAH1 | Inhibits |
| ASF1A | Induces |
| ASPH | Inhibits |
| ASXL2 | Induces |
| ATF3 | Inhibits |
| ATF6 | Induces |
| ATF7IP | Inhibits |
| ATG10 | Induces |
| ATG12 | Inhibits |
| ATG16L1 | Unclear |
| ATG4D | Inhibits |
| ATG5 | Inhibits |
| ATG7 | Inhibits |
| ATM | Inhibits |
| ATP6V0A2 | Inhibits |
| ATP6V0C | Inhibits |
| ATR | Induces |
| ATRAID | Induces |
| ATRX | Induces |
| ATXN10 | Induces |
| AURKA | Inhibits |
| AURKB | Inhibits |
| AXL | Induces |
| BAG3 | Inhibits |
| BAP1 | Induces |
| BAZ1A | Inhibits |
| BCL11B | Inhibits |
| BCL2 | Induces |
| BCL2L1 | Inhibits |
| BCL2L2 | Induces |
| BCL3 | Inhibits |
| BCL6 | Inhibits |
| BCLAF1 | Induces |
| BECN1 | Inhibits |
| BHLHE40 | Induces |
| BIN1 | Induces |
| BIRC5 | Inhibits |
| BLK | Induces |

| | |
|---|---|
| BLVRA | Inhibits |
| BMI1 | Inhibits |
| BMP4 | Induces |
| BMPR2 | Induces |
| BMS1 | Inhibits |
| BNIP3L | Induces |
| BRAF | Induces |
| BRCA1 | Inhibits |
| BRD4 | Inhibits |
| BRD7 | Induces |
| BRIP1 | Inhibits |
| BTG1 | Induces |
| BTG2 | Induces |
| BTG3 | Inhibits |
| BUB1 | Inhibits |
| BUB1B | Inhibits |
| CALR | Inhibits |
| CAPNS1 | Induces |
| CARF | Induces |
| CARM1 | Inhibits |
| CASP2 | Induces |
| CAV1 | Induces |
| CAVIN1 | Induces |
| CBS | Inhibits |
| CBX5 | Inhibits |
| CBX7 | Inhibits |
| CBX8 | Inhibits |
| CCL2 | Induces |
| CCN2 | Induces |
| CCN6 | Induces |
| CCNA2 | Inhibits |
| CCNB1 | Inhibits |
| CCND1 | Induces |
| CCND3 | Inhibits |
| CD28 | Inhibits |
| CD34 | Induces |
| CD40LG | Unclear |
| CD82 | Induces |
| CDC25A | Inhibits |

| | |
|---|---|
| CDC45 | Inhibits |
| CDC6 | Induces |
| CDC7 | Inhibits |
| CDCA2 | Inhibits |
| CDCA4 | Inhibits |
| CDH1 | Inhibits |
| CDK1 | Inhibits |
| CDK18 | Induces |
| CDK2 | Inhibits |
| CDK2AP1 | Inhibits |
| CDK4 | Inhibits |
| CDK5 | Induces |
| CDK5R1 | Induces |
| CDK6 | Inhibits |
| CDKN1A | Induces |
| CDKN1B | Induces |
| CDKN1C | Induces |
| CDKN2A | Induces |
| CDKN2AIP | Induces |
| CDKN2B | Induces |
| CEACAM1 | Induces |
| CEBPB | Induces |
| CEBPG | Inhibits |
| CENPA | Inhibits |
| CHAF1B | Inhibits |
| CHD5 | Induces |
| CHEK1 | Induces |
| CHEK2 | Induces |
| CHUK | Induces |
| CIP2A | Inhibits |
| CIT | Inhibits |
| CKAP2 | Inhibits |
| CKB | Induces |
| CLCA2 | Induces |
| CLPP | Inhibits |
| CLSPN | Inhibits |
| CLU | Inhibits |
| CNOT6 | Inhibits |
| CNOT6L | Inhibits |

| | |
|---|---|
| COX5B | Inhibits |
| CPEB1 | Induces |
| CPT1C | Inhibits |
| CREG1 | Induces |
| CRISPLD2 | Inhibits |
| CSNK1A1 | Inhibits |
| CSNK2A1 | Inhibits |
| CTH | Inhibits |
| CTNNAL1 | Inhibits |
| CTNNB1 | Induces |
| CTSB | Inhibits |
| CTSD | Inhibits |
| CUL4B | Inhibits |
| CUX1 | Inhibits |
| CXCL1 | Induces |
| CXCR2 | Induces |
| CYB5R3 | Inhibits |
| CYBB | Induces |
| CYP26A1 | Inhibits |
| DAO | Induces |
| DDAH2 | Inhibits |
| DDB1 | Inhibits |
| DDB2 | Induces |
| DDIT4 | Inhibits |
| DEK | Inhibits |
| DEPTOR | Inhibits |
| DGCR8 | Inhibits |
| DHCR24 | Induces |
| DHRS2 | Induces |
| DHX9 | Inhibits |
| DICER1 | Inhibits |
| DIDO1 | Inhibits |
| DIRAS3 | Inhibits |
| DKC1 | Inhibits |
| DLC1 | Induces |
| DMTF1 | Induces |
| DNMT1 | Inhibits |
| DNMT3A | Inhibits |
| DNMT3B | Inhibits |

| | |
|---|---|
| DOT1L | Inhibits |
| DPP4 | Induces |
| DPY30 | Inhibits |
| DTL | Inhibits |
| DUSP1 | Induces |
| DUSP16 | Inhibits |
| DUSP21 | Inhibits |
| DUSP3 | Inhibits |
| DUSP6 | Inhibits |
| DYRK1A | Induces |
| E2F1 | Induces |
| E2F3 | Inhibits |
| E2F7 | Induces |
| ECT2 | Inhibits |
| EEF1E1 | Induces |
| EGFR | Inhibits |
| EGLN1 | Induces |
| EGR2 | Induces |
| EHF | Induces |
| EHMT2 | Inhibits |
| EID3 | Induces |
| EIF2AK2 | Induces |
| EIF2AK3 | Inhibits |
| EIF4E | Unclear |
| EIF4EBP1 | Inhibits |
| EIF4G2 | Inhibits |
| ELAVL1 | Inhibits |
| ELOA | Inhibits |
| ENDOG | Inhibits |
| ENG | Induces |
| ENO1 | Inhibits |
| ENTPD7 | Induces |
| EP300 | Induces |
| EPAS1 | Inhibits |
| EPHA3 | Inhibits |
| EPHA5 | Inhibits |
| EPOR | Inhibits |
| ERBB2 | Induces |
| ERCC1 | Inhibits |

| | |
|---|---|
| ERRFI1 | Induces |
| ERVW-1 | Induces |
| ESPL1 | Inhibits |
| ESR1 | Inhibits |
| ESRRB | Induces |
| ETS1 | Induces |
| ETS2 | Induces |
| ETV6 | Induces |
| EWSR1 | Induces |
| EZH2 | Inhibits |
| FANCD2 | Induces |
| FASN | Induces |
| FASTK | Induces |
| FBXO22 | Induces |
| FBXO31 | Induces |
| FBXO4 | Induces |
| FBXO5 | Inhibits |
| FBXW11 | Inhibits |
| FDPS | Inhibits |
| FERMT1 | Inhibits |
| FERMT2 | Inhibits |
| FGF21 | Inhibits |
| FGFR1 | Induces |
| FGFR2 | Unclear |
| FGFR3 | Induces |
| FIS1 | Inhibits |
| FLT1 | Inhibits |
| FNTB | Inhibits |
| FOS | Inhibits |
| FOXA1 | Induces |
| FOXD1 | Unclear |
| FOXM1 | Inhibits |
| FOXO1 | Induces |
| FOXO3 | Inhibits |
| FOXO4 | Induces |
| FOXP1 | Inhibits |
| FOXP3 | Induces |
| FOXQ1 | Inhibits |
| FSCN1 | Inhibits |

| | |
|---|---|
| FXN | Inhibits |
| FXR1 | Inhibits |
| G6PD | Inhibits |
| GADD45G | Induces |
| GAPDH | Inhibits |
| GATA4 | Induces |
| GDF15 | Induces |
| GEMIN2 | Induces |
| GGCT | Inhibits |
| GJA1 | Induces |
| GKN1 | Induces |
| GLI1 | Inhibits |
| GMNN | Inhibits |
| GMPS | Inhibits |
| GNG11 | Induces |
| GNMT | Induces |
| GPC3 | Inhibits |
| GRIK2 | Induces |
| GRK4 | Induces |
| GRK6 | Induces |
| GRN | Inhibits |
| GRPR | Inhibits |
| GRSF1 | Inhibits |
| GSK3A | Inhibits |
| GSK3B | Inhibits |
| GTSE1 | Inhibits |
| HAS1 | Inhibits |
| HAUS4 | Inhibits |
| HBP1 | Induces |
| HDAC1 | Induces |
| HDAC2 | Inhibits |
| HDAC3 | Inhibits |
| HDAC4 | Inhibits |
| HDAC7 | Inhibits |
| HELLS | Inhibits |
| HEPACAM | Induces |
| HIF1A | Inhibits |
| HIPK2 | Induces |
| HIRA | Induces |

| | |
|---|---|
| HIVEP1 | Inhibits |
| HJURP | Inhibits |
| HK3 | Induces |
| HMGA1 | Induces |
| HMGA2 | Induces |
| HMGB1 | Inhibits |
| HMGB2 | Inhibits |
| HMGB3 | Inhibits |
| HMGCR | Inhibits |
| HNRNPA1 | Inhibits |
| HNRNPA3 | Inhibits |
| HNRNPAB | Inhibits |
| HNRNPC | Induces |
| HNRNPD | Inhibits |
| HOPX | Induces |
| HOXA9 | Inhibits |
| HRAS | Induces |
| HS2ST1 | Inhibits |
| HSF1 | Inhibits |
| HSP90AA1 | Inhibits |
| HSP90AB1 | Inhibits |
| HSPA1A | Inhibits |
| HSPA2 | Inhibits |
| HSPA5 | Inhibits |
| HSPA9 | Induces |
| HSPB1 | Inhibits |
| HTRA1 | Induces |
| HYOU1 | Inhibits |
| ID1 | Inhibits |
| IFI16 | Induces |
| IFNG | Induces |
| IGFBP1 | Induces |
| IGFBP3 | Induces |
| IGFBP5 | Induces |
| IGFBP7 | Induces |
| IKBKG | Induces |
| IL1A | Induces |
| IL1R1 | Induces |
| IL1RN | Unclear |

| | |
|---|---|
| IL24 | Inhibits |
| IL6 | Induces |
| ILF3 | Inhibits |
| ILK | Inhibits |
| IMMT | Inhibits |
| INCENP | Inhibits |
| ING1 | Induces |
| ING2 | Induces |
| ING5 | Induces |
| INPP4B | Inhibits |
| IRAK4 | Inhibits |
| IRF1 | Induces |
| IRF3 | Induces |
| IRF5 | Induces |
| IRF7 | Induces |
| ITGB3 | Induces |
| ITGB4 | Induces |
| ITPK1 | Induces |
| ITPKB | Induces |
| ITPR1 | Induces |
| ITPR2 | Induces |
| ITPR3 | Induces |
| ITSN2 | Induces |
| JAK2 | Induces |
| JPT1 | Inhibits |
| JUN | Inhibits |
| JUNB | Induces |
| KAT5 | Induces |
| KCNA1 | Induces |
| KCNJ12 | Inhibits |
| KDM1A | Inhibits |
| KDM2B | Inhibits |
| KDM4A | Unclear |
| KDM4C | Inhibits |
| KDM5B | Inhibits |
| KDM6B | Induces |
| KDR | Inhibits |
| KEAP1 | Induces |
| KIF11 | Inhibits |

| | |
|---|---|
| KIF20A | Inhibits |
| KIF2C | Inhibits |
| KIFC1 | Inhibits |
| KL | Inhibits |
| KLF4 | Induces |
| KNDC1 | Induces |
| KRT19 | Inhibits |
| KSR2 | Unclear |
| LAMP2 | Inhibits |
| LATS1 | Inhibits |
| LATS2 | Induces |
| LAYN | Induces |
| LBR | Inhibits |
| LCN2 | Inhibits |
| LEO1 | Unclear |
| LGALS3 | Inhibits |
| LIMA1 | Inhibits |
| LIMK1 | Induces |
| LIN52 | Unclear |
| LIN54 | Inhibits |
| LIN9 | Inhibits |
| LMNA | Unclear |
| LMNB1 | Inhibits |
| LNCTAM34A | Inhibits |
| LOX | Inhibits |
| LOXL2 | Inhibits |
| LPAR1 | Induces |
| LPAR3 | Inhibits |
| LRRK2 | Unclear |
| LY6D | Induces |
| MAD1L1 | Induces |
| MAD2L1 | Inhibits |
| MAEL | Inhibits |
| MAF | Inhibits |
| MAGEA2 | Inhibits |
| MAGOHB | Inhibits |
| MAP2K1 | Induces |
| MAP2K2 | Induces |
| MAP2K3 | Inhibits |

| | |
|---|---|
| MAP2K6 | Induces |
| MAP2K7 | Induces |
| MAP3K14 | Induces |
| MAP3K5 | Induces |
| MAP3K6 | Induces |
| MAP3K7 | Induces |
| MAP4K1 | Inhibits |
| MAPK1 | Induces |
| MAPK12 | Induces |
| MAPK14 | Induces |
| MAPK9 | Inhibits |
| MAPKAPK5 | Induces |
| MARCHF5 | Inhibits |
| MARCKS | Induces |
| MAST1 | Induces |
| MATK | Induces |
| MAVS | Induces |
| MCAM | Inhibits |
| MCL1 | Inhibits |
| MCM3AP | Inhibits |
| MCM7 | Inhibits |
| MCRS1 | Induces |
| MCU | Induces |
| MDC 1.00 | Inhibits |
| MDH1 | Inhibits |
| MDK | Inhibits |
| MDM2 | Inhibits |
| MDP1 | Induces |
| ME1 | Inhibits |
| ME2 | Inhibits |
| MECP2 | Inhibits |
| MED12 | Inhibits |
| MEF2A | Inhibits |
| MEIS2 | Inhibits |
| MEN1 | Induces |
| MEOX1 | Induces |
| MET | Inhibits |
| METTL14 | Induces |
| MIF | Inhibits |

| | |
|---|---|
| MINK1 | Induces |
| MITF | Unclear |
| MKRN1 | Inhibits |
| MME | Induces |
| MMP7 | Inhibits |
| MMP9 | Inhibits |
| MNX1 | Induces |
| MOB3A | Induces |
| MORC3 | Induces |
| MORF4L1 | Inhibits |
| MOS | Induces |
| MRTFA | Inhibits |
| MRTFB | Inhibits |
| MSN | Inhibits |
| MST1 | Induces |
| MT1G | Induces |
| MTDH | Inhibits |
| MTHFD2 | Inhibits |
| MTOR | Inhibits |
| MUC4 | Inhibits |
| MUS81 | Inhibits |
| MVK | Induces |
| MVP | Induces |
| MXD4 | Induces |
| MYBBP1A | Inhibits |
| MYBL2 | Inhibits |
| MYC | Inhibits |
| MYCN | Inhibits |
| MYD88 | Inhibits |
| MYLK | Inhibits |
| NACC1 | Inhibits |
| NADK | Induces |
| NAMPT | Inhibits |
| NANOG | Inhibits |
| NBN | Inhibits |
| NBR1 | Inhibits |
| NCAPG2 | Inhibits |
| NCAPH2 | Induces |
| NDRG1 | Induces |

| | |
|---|---|
| NDST2 | Induces |
| NEDD4 | Inhibits |
| NEK1 | Inhibits |
| NEK2 | Inhibits |
| NEK4 | Induces |
| NEK6 | Inhibits |
| NEK9 | Inhibits |
| NF1 | Inhibits |
| NF2 | Induces |
| NFE2L2 | Inhibits |
| NFKB2 | Inhibits |
| NFKBIA | Induces |
| NHEJ1 | Inhibits |
| NINJ1 | Induces |
| NIPA2 | Inhibits |
| NLK | Inhibits |
| NLRX1 | Induces |
| NOLC1 | Induces |
| NOTCH1 | Induces |
| NOTCH3 | Induces |
| NOX1 | Induces |
| NOX4 | Induces |
| NPM1 | Inhibits |
| NQO1 | Induces |
| NR1H2 | Inhibits |
| NR2E1 | Unclear |
| NRAS | Induces |
| NRF1 | Inhibits |
| NRSN2 | Induces |
| NSUN2 | Inhibits |
| NTN4 | Inhibits |
| NUAK1 | Induces |
| NUAK2 | Inhibits |
| NUDT5 | Inhibits |
| NUTF2 | Inhibits |
| OGG1 | Inhibits |
| OGT | Induces |
| OPA1 | Inhibits |
| ORAI1 | Induces |

| | |
|---|---|
| ORC1 | Inhibits |
| OTX2 | Induces |
| OXTR | Inhibits |
| P2RY14 | Inhibits |
| P3H1 | Inhibits |
| PAK2 | Induces |
| PAK4 | Induces |
| PAPSS2 | Inhibits |
| PARK7 | Inhibits |
| PARP1 | Inhibits |
| PATZ1 | Inhibits |
| PAX8 | Inhibits |
| PBRM1 | Induces |
| PCGF2 | Induces |
| PDCD10 | Induces |
| PDCD11 | Inhibits |
| PDCD4 | Inhibits |
| PDGFB | Induces |
| PDIK1L | Induces |
| PDPK1 | Induces |
| PDZD2 | Induces |
| PEA15 | Induces |
| PEBP1 | Induces |
| PELP1 | Inhibits |
| PES 1.00 | Inhibits |
| PEX19 | Induces |
| PGR | Induces |
| PHB | Induces |
| PHB2 | Inhibits |
| PHGDH | Inhibits |
| PI4KB | Induces |
| PIK3C2A | Unclear |
| PIK3CA | Unclear |
| PIK3R5 | Induces |
| PIM1 | Induces |
| PIN1 | Induces |
| PINK1 | Inhibits |
| PINX1 | Induces |
| PIR | Inhibits |

| | |
|---|---|
| PITX1 | Induces |
| PLA2G2A | Induces |
| PLA2R1 | Induces |
| PLD2 | Inhibits |
| PLK1 | Inhibits |
| PML | Induces |
| PMVK | Induces |
| PNPT1 | Induces |
| PON1 | Inhibits |
| POT1 | Inhibits |
| POU3F1 | Induces |
| POU5F1 | Induces |
| PPARG | Induces |
| PPARGC1A | Inhibits |
| PPIB | Inhibits |
| PPM1B | Inhibits |
| PPM1D | Inhibits |
| PPP1R13B | Inhibits |
| PPP2R1A | Induces |
| PPP2R5A | Induces |
| PRKAA2 | Inhibits |
| PRKCD | Induces |
| PRKCH | Induces |
| PRKD1 | Induces |
| PRKD2 | Inhibits |
| PRKDC | Inhibits |
| PRKN | Inhibits |
| PRMT1 | Inhibits |
| PRMT6 | Inhibits |
| PRODH | Induces |
| PROX1 | Induces |
| PRPF19 | Inhibits |
| PSMA2 | Inhibits |
| PSMA5 | Inhibits |
| PSMB1 | Inhibits |
| PSMB5 | Inhibits |
| PSMD14 | Inhibits |
| PTEN | Inhibits |
| PTGS2 | Induces |

| | |
|---|---|
| PTK2 | Inhibits |
| PTPN1 | Unclear |
| PTPN6 | Inhibits |
| PTTG1 | Induces |
| PURB | Induces |
| PYGL | Inhibits |
| RACGAP1 | Inhibits |
| RACK1 | Inhibits |
| RAD21 | Inhibits |
| RAD23B | Inhibits |
| RAD51C | Inhibits |
| RAF1 | Induces |
| RAN | Inhibits |
| RANBP9 | Inhibits |
| RAP1GAP | Induces |
| RAPGEF4 | Inhibits |
| RARB | Induces |
| RASSF1 | Induces |
| RASSF4 | Inhibits |
| RASSF5 | Induces |
| RB1 | Induces |
| RBBP4 | Inhibits |
| RBL1 | Induces |
| RBL2 | Induces |
| RBM38 | Induces |
| RBM39 | Inhibits |
| RBP1 | Induces |
| RBP2 | Inhibits |
| RBPJ | Induces |
| RBX1 | Inhibits |
| RCC1 | Inhibits |
| RECK | Inhibits |
| RECQL4 | Inhibits |
| RELA | Induces |
| RELB | Induces |
| RHOA | Inhibits |
| RNASEH2A | Inhibits |
| RNASEH2B | Inhibits |
| RNASEL | Induces |

| | |
|---|---|
| ROMO1 | Induces |
| RPL11 | Induces |
| RPS14 | Induces |
| RPS6KB1 | Induces |
| RPS9 | Inhibits |
| RPTOR | Induces |
| RRAD | Inhibits |
| RRAS2 | Inhibits |
| RRM1 | Inhibits |
| RRM2 | Inhibits |
| RRM2B | Inhibits |
| RRP8 | Induces |
| RTN4 | Induces |
| RUNX1 | Induces |
| RUVBL2 | Inhibits |
| S100A6 | Inhibits |
| SALL1 | Induces |
| SAMHD1 | Inhibits |
| SAT2 | Inhibits |
| SDC1 | Inhibits |
| SELENBP1 | Induces |
| SELENOH | Inhibits |
| SENP1 | Inhibits |
| SENP7 | Inhibits |
| SERPINB2 | Induces |
| SERPINE1 | Induces |
| SETD1A | Inhibits |
| SFN | Induces |
| SFRP1 | Induces |
| SGK1 | Inhibits |
| SIAH1 | Induces |
| SIK1 | Induces |
| SIN3B | Induces |
| SIRT1 | Inhibits |
| SIRT2 | Inhibits |
| SIRT3 | Inhibits |
| SIRT6 | Inhibits |
| SIRT7 | Inhibits |
| SIX1 | Inhibits |

| | |
|---|---|
| SIX6 | Unclear |
| SKP2 | Inhibits |
| SLC13A3 | Induces |
| SLC16A7 | Inhibits |
| SLC25A5 | Inhibits |
| SLC31A2 | Induces |
| SLC52A1 | Inhibits |
| SLC5A2 | Induces |
| SMAD1 | Induces |
| SMAD2 | Inhibits |
| SMAD3 | Induces |
| SMAD5 | Induces |
| SMAD6 | Inhibits |
| SMARCA2 | Inhibits |
| SMARCA4 | Inhibits |
| SMARCB1 | Induces |
| SMARCD1 | Inhibits |
| SMC1A | Inhibits |
| SMC2 | Inhibits |
| SMG1 | Inhibits |
| SMURF2 | Induces |
| SNAI1 | Inhibits |
| SOCS1 | Induces |
| SOD1 | Inhibits |
| SOD2 | Induces |
| SORBS2 | Induces |
| SOX1 | Induces |
| SOX2 | Induces |
| SOX4 | Inhibits |
| SOX5 | Induces |
| SP1 | Induces |
| SPAG9 | Inhibits |
| SPARC | Induces |
| SPHK1 | Inhibits |
| SPI1 | Induces |
| SPIN1 | Induces |
| SPOP | Induces |
| SRC | Inhibits |
| SREBF1 | Induces |

| | |
|---|---|
| SRF | Inhibits |
| SRSF1 | Induces |
| SRSF2 | Inhibits |
| SRSF3 | Inhibits |
| SSX2 | Induces |
| STAG2 | Induces |
| STAT1 | Induces |
| STAT3 | Induces |
| STAT5A | Induces |
| STAT5B | Induces |
| STAT6 | Induces |
| STIM1 | Unclear |
| STK32C | Induces |
| STK4 | Induces |
| STK40 | Induces |
| STN 1.00 | Inhibits |
| STUB1 | Inhibits |
| SUMO2 | Induces |
| SUMO3 | Induces |
| SUPT5H | Inhibits |
| SUV39H1 | Inhibits |
| SUZ12 | Inhibits |
| SYK | Induces |
| SYT1 | Inhibits |
| SYT7 | Inhibits |
| TACC3 | Inhibits |
| TAGLN | Induces |
| TBK1 | Inhibits |
| TBPL1 | Induces |
| TBX2 | Inhibits |
| TBX3 | Inhibits |
| TEAD1 | Inhibits |
| TEAD4 | Inhibits |
| TERF2 | Inhibits |
| TERT | Inhibits |
| TFDP1 | Inhibits |
| TFG | Inhibits |
| TGFB1I1 | Induces |
| TGFB2 | Inhibits |

| | |
|---|---|
| TGFBI | Induces |
| TGFBR1 | Inhibits |
| TGFBR2 | Unclear |
| THRB | Unclear |
| TIGAR | Induces |
| TIMELESS | Inhibits |
| TLR10 | Induces |
| TLR2 | Induces |
| TLR3 | Induces |
| TLR4 | Inhibits |
| TLR8 | Inhibits |
| TMEM9B | Induces |
| TNFSF13 | Inhibits |
| TNFSF15 | Induces |
| TOM1 | Induces |
| TOP 1.00 | Induces |
| TOP3A | Induces |
| TOPBP1 | Inhibits |
| TP53 | Induces |
| TP53BP1 | Induces |
| TP53BP2 | Induces |
| TP53I3 | Inhibits |
| TP53INP1 | Induces |
| TP63 | Induces |
| TPP1 | Inhibits |
| TPR | Inhibits |
| TPX2 | Inhibits |
| TRA2B | Inhibits |
| TRDMT1 | Inhibits |
| TRIM28 | Induces |
| TRPM7 | Inhibits |
| TRPM8 | Inhibits |
| TRRAP | Inhibits |
| TSC22D1 | Inhibits |
| TTK | Inhibits |
| TWIST1 | Inhibits |
| TWIST2 | Inhibits |
| TXN | Inhibits |
| TXNIP | Induces |

| | |
|---|---|
| TYK2 | Induces |
| TYMS | Inhibits |
| UBE2C | Inhibits |
| UBE2I | Inhibits |
| UBE2N | Inhibits |
| UBE2V1 | Inhibits |
| UBE2V2 | Inhibits |
| UBE3A | Inhibits |
| UBTD1 | Induces |
| UHRF1 | Inhibits |
| ULK3 | Induces |
| USP1 | Inhibits |
| USP28 | Induces |
| UTP6 | Inhibits |
| VCAN | Induces |
| VDR | Inhibits |
| VEGFA | Inhibits |
| VENTX | Induces |
| WEE1 | Inhibits |
| WIF1 | Induces |
| WIPI1 | Inhibits |
| WNT16 | Induces |
| WNT2 | Inhibits |
| WNT5A | Induces |
| WNT7B | Inhibits |
| WRN | Inhibits |
| WSB1 | Inhibits |
| WT1 | Inhibits |
| WWP1 | Inhibits |
| XAF1 | Induces |
| XIAP | Inhibits |
| XPC | Inhibits |
| XPO1 | Induces |
| YAP1 | Inhibits |
| YBX1 | Inhibits |
| YEATS4 | Inhibits |
| YPEL3 | Induces |
| YWHAB | Inhibits |
| YWHAZ | Inhibits |

| | |
|---|---|
| YY1 | Inhibits |
| ZCCHC10 | Induces |
| ZDHHC3 | Inhibits |
| ZEB1 | Inhibits |
| ZFP36 | Induces |
| ZFX | Inhibits |
| ZMAT3 | Inhibits |
| ZMPSTE24 | Inhibits |
| ZMYND11 | Inhibits |
| ZNF148 | Inhibits |
| ZNF207 | Inhibits |
| ZNF217 | Inhibits |

Table S2. the list of the primers used in this study

| Primer Name | Sequence (5'to3') |
|---|---|
| BTG3(F) | AAGAACGAAATTGCGGCTGTT |
| BTG3(R) | CATCGGGATCAACTCTCTGAAAC |
| LOX(F) | TCTTCTGCTGCGTGACAACC |
| LOX(R) | GAGAAACCAGCTTGGAACCAG |
| FOXD1(F) | CTTCTCCATCGAGAGCCTCAT |
| FOXD1(R) | CTGTCCCTTGGTGCAGAGTC |
| GJA1(F) | ACAGCGGTTGAGTCAGCTTG |
| GJA1(R) | GAGAGATGGGGAAGGACTTGT |
| Kim-1(F) | ACATATCGTGGAATCACAACGAC |
| Kim-1(R) | ACAAGCAGAAGATGGGCATTG |

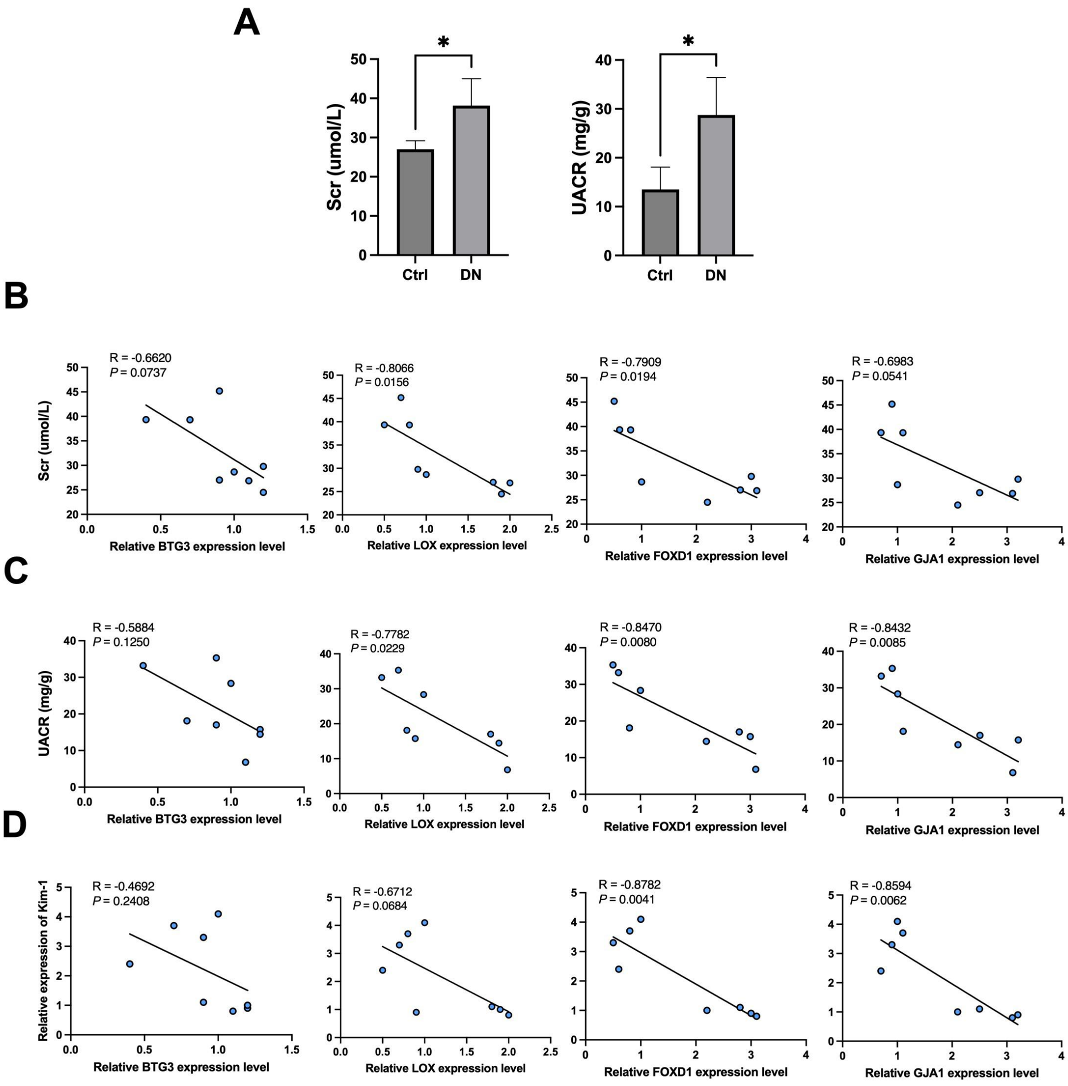


Figure S1. Correlation analysis between renal function indicators and the protein expression levels of feature genes. (A) Comparisons of expression levels of renal function indicators (Scr and uACR). (B) Correlation analysis between expression levels of feature genes and Scr. (C) Correlation analysis between expression levels of feature genes and uACR. (D) Correlation analysis between expression levels of feature genes and Kim-1. Results were presented as mean ± SD., $^{*}p < 0.05$, $^{***}p < 0.001$.
